# Extended antibiotic prophylaxis beyond 24 h after pancreatic surgery: prevalence and risk factors

**DOI:** 10.3389/fpubh.2026.1727898

**Published:** 2026-05-28

**Authors:** Zhanjie Li, Can Luo, Chuanlong Zhu, Xiaoju Ma

**Affiliations:** 1 Department of Infection Control, The First Affiliated Hospital with Nanjing Medical University, Nanjing, Jiangsu, China; 2 Department of Epidemiology and Health Statistics, School of Public Health, Fudan University, Shanghai, China; 3 Institute of Hospital Reform and Development of China, Nanjing University, Nanjing, Jiangsu, China; 4 Department of Pharmacy, The First Affiliated Hospital with Nanjing Medical University, Nanjing, Jiangsu, China; 5 Department of Infectious Disease, The First Affiliated Hospital with Nanjing Medical University, Nanjing, Jiangsu, China; 6 Department of Hospital Acquired Infection Control and Public Health Management, The Seventh Affiliated Hospital, Sun Yat-sen University, Shenzhen, Guangdong, China

**Keywords:** antibiotic prophylaxis, antimicrobial stewardship, guideline adherence, pancreatic surgery, surgical site infection

## Abstract

**Objective:**

To evaluate the compliance with antibiotic prophylaxis (AP) guidelines after pancreatic surgery, identify predictors of extended AP usage (>24 h), and assess the association between extended AP and surgical site infection (SSI).

**Methods:**

A retrospective cohort study was conducted involving 1,073 patients who underwent pancreatic surgery at the First Affiliated Hospital with Nanjing Medical University between January and December 2022. Based on the strict intent-of-use definition in the electronic medical record, patients were categorized into the AP > 24 h group (*n* = 135) and the Non-AP > 24 h group (*n* = 938). Univariate and multivariate logistic regression analyses were performed to identify factors associated with AP > 24 h. Additionally, a hierarchical modeling approach was employed to evaluate the independent association between extended AP and the risk of SSI.

**Results:**

Of the 1,073 patients, 12.58% (135/1,073) received AP > 24 h. The cumulative days of postoperative AP ranged from 1 to 24, with a mean of 6.24 ± 4.09 days. While 93.20% of surgeries were performed in specialized pancreatic centers, 6.80% were performed in non-pancreatic centers. Multivariate analysis identified that surgery performed in non-pancreatic centers was an independent predictor associated with AP > 24 h (OR = 2.61, 95%CI = 1.04–6.54; *p* = 0.041). Crucially, in the fully adjusted model, extending AP beyond 24 h was not associated with a lower risk of SSI (OR = 1.67, 95% CI: 0.997–2.79; *p* = 0.053).

**Conclusion:**

A substantial gap between guidelines and clinical practice persists, with 12.58% of patients receiving extended AP. Surgery performed in non-pancreatic centers was identified as a significant predictor for non-adherence, with the odds of extended use being 2.61 times that of specialized centers. Given that prolonged AP was not associated with reduced SSI rates, targeted stewardship interventions are urgently needed to standardize protocols, particularly in non-specialized departments, to avoid unnecessary antibiotic exposure.

## Introduction

Surgical site infection (SSI) is one of the most common health care-associated infections (HCAIs) in medical institutions, accounting for nearly 20% of all HCAIs (1), and SSI is associated with a prolongation of hospitalization by 7–11 days and a 2-to-11-fold increase in mortality (2). The use of perioperative antibiotic prophylaxis (AP) is an important strategy for preventing SSI. Studies have shown that standardized AP can reduce the risk of SSI by approximately 50% (3).

Pancreatic surgery, where incisions are mostly type II, is one of the most complex and technically challenging procedures in the field of general surgery (4). Despite improvements in medical and surgical management, pancreatic surgery still has a high complication rate. In particular, postoperative infections and postoperative pancreatic fistulas (POPF) frequently occur after pancreaticoduodenectomy and may lead to prolonged hospitalization, delayed recovery and even death (5). Lin et al. (6) reviewed Chinese studies on SSI rates and reported that in China, the median SSI rate after pancreatic surgery was 6.67%, and the co-morbidity rate reached 11.22%, which is second only to that of colorectal surgery (12.54%).

The correct administration of antimicrobial drugs in terms of dose, timing, route and duration is critical for the optimal use of AP (7). Although AP plays a key role in the prevention of SSI, excessively prolonging the administration of antimicrobials cannot further enhance their preventive effect. Instead, it may destroy the normal flora in the human body, eventually leading to drug resistance and an increased risk of secondary infections (8, 9). According to relevant regulatory guidelines in China and abroad (10–12), except for cardiac surgery, the duration of postoperative prophylaxis for clean surgery should not exceed 24 h. For clean-contaminated surgery and contaminated surgery, the postoperative prophylaxis should also not exceed 24 h, unless it is necessary to extend to 48 h. Although there are some minor differences, the current requirements follow the same basic principle: it is considered inappropriate to prolong prophylaxis for more than 24 h after surgery (13), and even for patients with multidrug-resistant gram-negative bacteria (MDR-GNB) colonization, it is also recommended that AP should be routinely discontinued within 24 h after surgery (14). However, these recommendations are not fully followed. One point prevalence study (PPS) revealed a prolonged treatment duration (>1 day) in more than half of surgical prophylaxis treatments in European countries (15).

Perioperative AP is a primary indicator of global antibiotic consumption (13). It has been reported that AP accounts for 6–33.33% of all antimicrobial drugs used in hospitals worldwide (4, 13). AP is also one of the most common types of antibacterial treatment in Europe, which has led to the increased consumption of antimicrobial drugs and the consequent emergence of antibacterial resistance (15). Recent studies on AP have focused on the prophylaxis use rate for type I incisions (clean surgery), the rate of postoperative prophylaxis use >24 h (16), and the timing of preoperative AP use (e.g., 0–30 min or 30–60 min prior to skin incision) (1). There are very few studies on postoperative AP time for type II incisions (clean-contaminated surgery and contaminated surgery).

To address this research gap, this study investigated the status of AP administration in patients undergoing pancreatic surgery. We aimed to characterize AP patterns, evaluate the prevalence of extended postoperative AP (>24 h), and identify factors associated with non-adherence.

## Materials and methods

### Study design and participants

This was a retrospective cohort study conducted at the First Affiliated Hospital with Nanjing Medical University. We reviewed the clinical data of all patients who underwent pancreatic surgery between January 2022 and December 2022.

**Figure 1:**
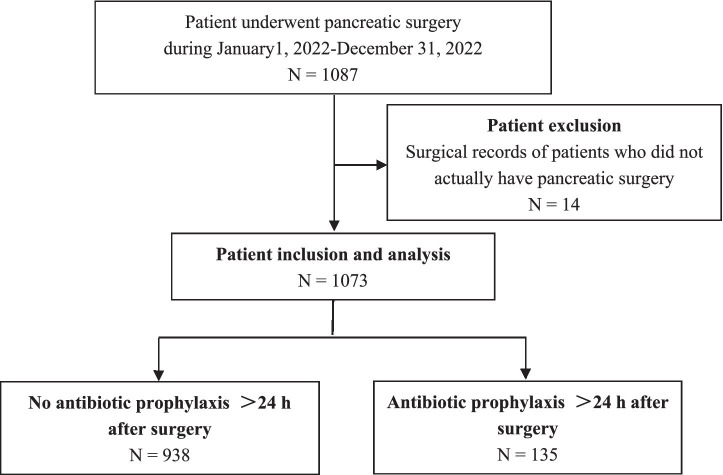
Flowchart of patient exclusion and inclusion.

The inclusion criteria were patients undergoing pancreatic surgery. The exclusion criteria were patients who did not undergo pancreatic surgery after laparotomy (e.g., unresectable tumors found intraoperatively). A total of 1,087 patients were initially identified; after excluding 14 cases, 1,073 patients were ultimately included in the analysis. Based on the duration of antibiotic prophylaxis, the cohort was divided into the AP > 24 h after surgery group (*n* = 135) and the Non-AP > 24 h after surgery group (*n* = 938), as detailed in Figure 1. The surgeries were performed in the pancreatic center (an independent specialized department) and non-pancreatic centers (including hepatobiliary, gastric, and colorectal surgery departments). The Pancreatic Center is a dedicated surgical unit exclusively performing pancreatic-related procedures with specialized expertise and infrastructure. Non-pancreatic centers refer to other surgical departments that perform pancreatic surgery occasionally as an ancillary component.

### Data collection and definitions

#### Data collection

Data were collected retrospectively using the Real-time Nosocomial Infection Surveillance System (RT-NISS) (Xinglin Technology, Hangzhou, China). This system automatically extracts demographic, surgical, and pharmacological, microbiological identification, and antimicrobial susceptibility data from the Hospital Information System (HIS).

#### Definition of antibiotic prophylaxis (AP)

To ensure the accuracy of the analysis, strict criteria were applied to distinguish AP from therapeutic use based on the physician’s prespecified “intent of use” in the electronic medical record system at the time of prescription. Only orders explicitly recorded as “prophylaxis” were defined as AP, whereas antibiotics recorded as “treatment” (e.g., for suspected or confirmed infections, or gross intraoperative contamination) were considered therapeutic and excluded from the calculation of AP duration.

#### Postoperative time period definition

**Figure 2:**
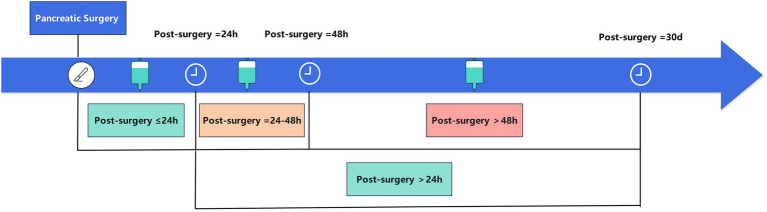
Postoperative time period definition.

Postoperative ≤24 h was defined as within 24 h after the end of surgery, 24–48 h was defined as 24–48 h after the end of surgery, and >48 h was defined as beyond 48 h after the end of surgery (Figure 2).

#### Postoperative AP rate and cumulative days

According to the *Chinese Hospital infection surveillance standards* (WS/T 312-2023) (17), the postoperative AP rate = number of patients who used AP after surgery/number of patients undergoing similar surgeries during the same period * 100%. Cumulative days of AP 24 h after surgery was the sum of the number of days of AP from 24 h to 30 d or discharge (patients discharged in less than 30 days) after surgery.

#### Classification of antimicrobial drugs

In this study, the third-generation cephalosporins included ceftizoxime, cefodizime and ceftriaxone; the second-generation cephalosporins included cefuroxime; the oxacephem included lanocephem; the cephalosporinase inhibitor combination preparations included cefoperazone-sulbactam and ceftriaxone-tazobactam; the quinolones included moxifloxacin and levofloxacin; the antifungal drug was fluconazole; the anti-anaerobic drug was morinidazole; the carbapenem was meropenem; and the drug classified as “other” was linezolid.

#### Variable selection

To identify independent risk factors for the AP > 24 h after surgery group, we analyzed variables based on clinical relevance and literature review. These variables included (1) basic variables, such as sex, age, and diabetes history; (2) surgery-related variables, such as surgical category (pancreaticoduodenectomy, distal pancreatectomy, other surgeries), surgical approach (non-endoscopic surgery, endoscopic surgery), duration of surgery (h), emergency surgery or not, American Society of Anaesthesiologists (ASA) score (1–2 points, 3–4 points), and National Nosocomial Infections Surveillance System (NNIS) score (0–1 point, 2–3 points), type of incision (type II incision, type III incision), amount of intraoperative blood loss (mL), Surgeon identity categorized as Doctor A–F and others, whether the intraoperative addition of antimicrobial drugs was standardized (required but no redosing, required and redosing, or not required), surgical departments (pancreatic center or non-pancreatic centers), preoperative endoscopic retrograde cholangiopancreatography (ERCP) (yes or no), intraoperative placement of biliary or pancreatic duct stents (yes or no), and postoperative occurrence of surgical site infection (SSI) (yes or no). The criteria for the intraoperative addition of antimicrobial drugs were that the operative duration was ≥3 h or the amount of intraoperative blood loss was ≥1,500 mL (11).

### Statistical analysis

WPS software (version 2023) was used to summarize the data. Normally distributed measurement data were expressed as the mean ± standard deviation ( $\bar{x}\pm s$ ), and an independent samples t test was used; count data were expressed as [*n* (%)], and the *x^2^* test was performed. To identify independent risk factors for AP > 24 h after pancreatic surgery, variables with *p* < 0.05 in the univariate analysis were included in the multivariate logistic regression analysis. Additionally, to evaluate the independent association between extended AP and SSI, a hierarchical modeling approach was employed, including an unadjusted model, Model 1 (adjusted for demographic characteristics), and Model 2 (fully adjusted for surgical and clinical variables). The results were expressed as odds ratios (ORs) with 95% confidence intervals (CIs), and a two-sided *p* < 0.05 was considered statistically significant. Statistical analyses were performed using SPSS version 25.0 (IBM Corp., Armonk, NY, USA), R software version 4.2.0 (R Core Team), and EmpowerStats,^1^ while figures were generated using GraphPad Prism 9.5.08 (San Diego, CA, USA) and EdrawMax (Wondershare, Hangzhou, China).

## Results

### AP after pancreatic surgery

**Figure 3:**
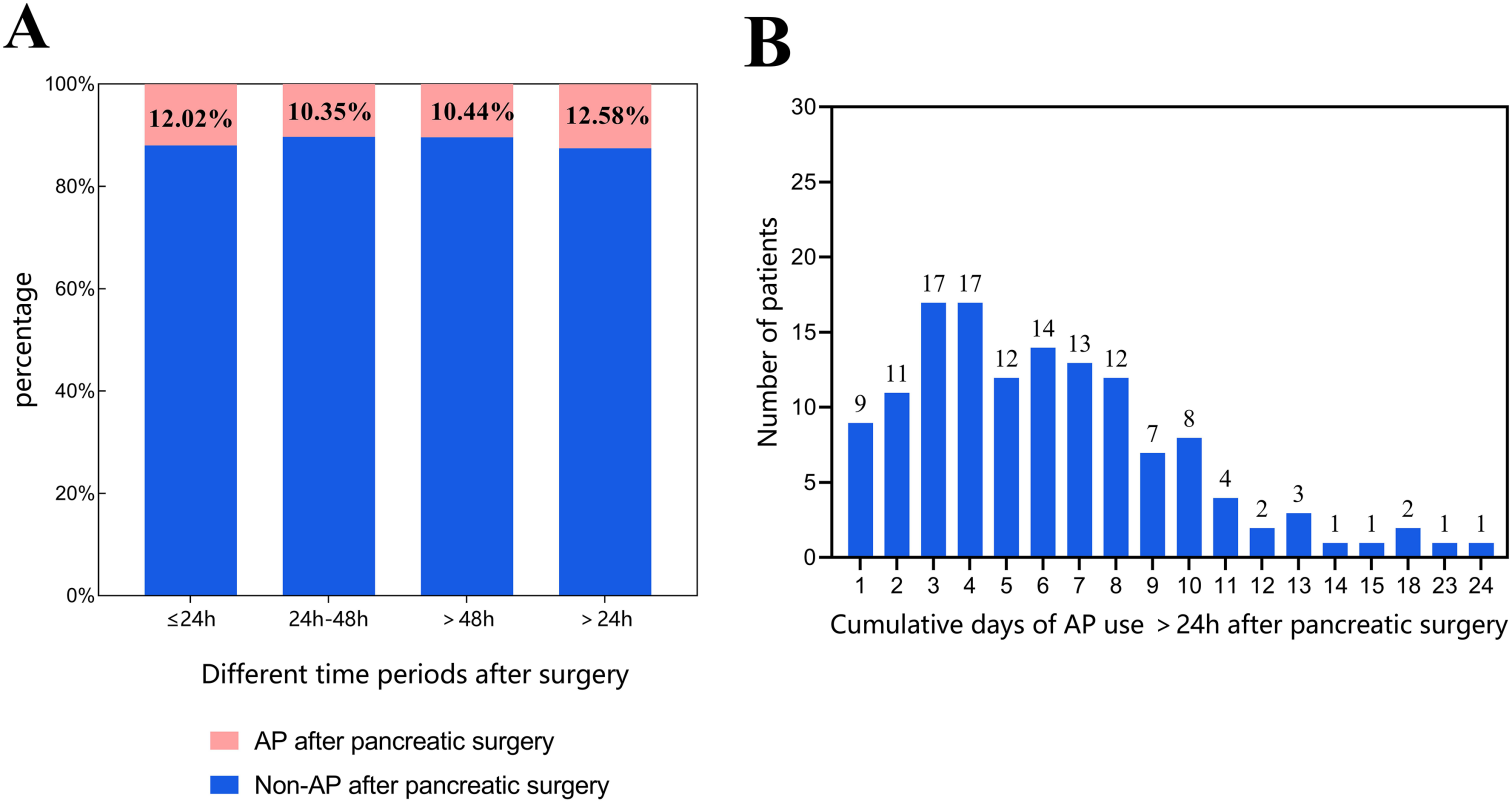
AP use after pancreatic surgery (*n* = 135). **(A)** AP use at different time periods after pancreatic surgery (*n* = 1,073). **(B)** Distribution of cumulative days of AP use 24 h after pancreatic surgery.

The rate of AP ≤ 24 h after pancreatic surgery was 12.02% (129/1,073); and the rate of AP > 24 h was 12.58% (135/1,073), and that for 24–48 h after surgery and >48 h after surgery was 10.34% (111/1,073) and 10.44% (112/1,073), respectively, as shown in Figure 3A. For the cumulative days of drug use, the minimum was 1 day, the maximum was 24 days, 1–8 days was most common, and the average was 6.24 ± 4.09 days, as shown in Figure 3B. Specifically, 1–3 days accounted for 27.41% (37/135), 4–7 days for 41.48% (56/135), 8–14 days for 27.41% (37/135), and ≥15 days for 3.70% (5/135).

### Distribution of AP types at different time periods after pancreatic surgery

In the 1,073 patients included in the study, the antimicrobial drugs used prophylactically for ≤24 h, 24–48 h and >48 h after pancreatic surgery were predominantly third-generation cephalosporins and cephalosporinase inhibitor combination preparations, with no statistically significant difference (*p >* 0.05) (Table 1).

**Table 1:** Distribution of AP types at different time periods after pancreatic surgery.

AP types	Time periods after pancreatic surgery		
	≤24 h (*n* = 129)	24–48 h (*n* = 111)	>48 h (*n* = 112)
Third-generation cephalosporins	85 (65.89)	74 (66.67)	65 (58.04)
Cephalosporinase inhibitor combination preparations	33 (25.58)	27 (24.32)	28 (25.00)
Second-generation cephalosporins	7 (5.43)	8 (7.21)	9 (8.04)
Oxacephem	3 (2.33)	3 (2.70)	5 (4.46)
Anti-anaerobic drug	2 (1.55)	1 (0.90)	0 (0)
Antifungal drug	1 (0.78)	2 (1.80)	5 (4.46)
Quinolones	1 (0.78)	1 (0.90)	6 (5.36)
Carbapenem	0 (0)	0 (0)	2 (1.79)
Other	0 (0)	0 (0)	1 (1.79)
Statistic	19.064		
*p*	0.265		

The three time periods (≤24 h, 24–48 h, >48 h) are not mutually exclusive. A patient is counted in each time period during which postoperative AP was administered; therefore, the column counts do not sum to the total number of patients.

### The clinical characteristics of patients receiving AP > 24 h after pancreatic surgery

The average age of the 1,073 patients undergoing pancreatic surgery was (59.93 ± 13.35) years. The proportion of males and females was 56.01 and 43.99%, respectively. Most procedures (93.20%) were performed in pancreatic centers, whereas 6.80% were performed in non-pancreatic centers. Compared with patients who did not receive the AP > 24 h after surgery, patients who received AP were older (63.22 ± 13.29 vs. 59.46 ± 13.30), had higher ASA scores (3–4 points: 31.11% vs. 20.26%), greater NNIS scores (2–3 points: 23.70% vs. 14.71%) and higher in non-pancreatic centers (21.48% vs. 4.69%). The incidence of SSI was higher in patients who received AP beyond 24 h than in those who did not (17.78% vs. 11.18%, *p* < 0.01). All differences were statistically significant (all *p* < 0.05), as shown in Table 2. In contrast, there were no significant differences between the two groups in the proportions of patients who underwent preoperative ERCP or intraoperative placement of biliary or pancreatic duct stents (both *p* > 0.05).

**Table 2:** Characteristics of 1,073 patients undergoing pancreatic surgery in the AP >24 h after surgery and non-AP >24 h after surgery groups.

**Characteristic**	**Total** **(*n* = 1073)**	**AP>24 h** **after surgery** **(*n* = 135)**	**Non-AP>24 h** **after surgery** **(*n* = 938)**	**Statistic**	***P* value**
**Age (years)**	59.93 ± 13.35	63.22 ± 13.29	59.46 ± 13.30	9.464	0.002
**Sex**				0.661	0.416
Male	601 (56.01)	80 (59.26)	521 (55.54)		
Female	472 (43.99)	55 (40.74)	417 (44.46)		
**Diabetes**				0.204	0.651
No	889 (82.85)	110 (81.48)	779 (83.05)		
Yes	184 (17.15)	25 (18.52)	159 (16.95)		
**Surgical category**				1.154	0.562
Pancreaticoduodenectomy	542 (50.51)	74 (54.81)	468 (49.89)		
Distal pancreatectomy	345 (32.15)	40 (29.63)	305 (32.52)		
Others	186 (17.33)	21 (15.56)	165 (17.59)		
**Surgical approach**				0.557	0.455
Non-endoscopic surgery	932 (86.86)	120 (88.89)	812 (86.57)		
Endoscopic surgery	141 (13.14)	15 (11.11)	126 (13.43)		
**Surgical time (h)**	4.00 ± 1.55	4.16 ± 1.64	3.97 ± 1.53	1.863	0.308
**Emergency**				0.015	0.904
No	1015(94.59)	128 (94.81)	887 (94.56)		
Yes	58 (5.41)	7 (5.19)	51 (5.44)		
**ASA score**				7.932	0.004
1-2 points	841 (78.38)	93 (68.89)	748 (79.74)		
3-4 points	232 (21.62)	42 (31.11)	190 (20.26)		
**NNIS score**				7.156	0.007
0-1 point	903 (84.16)	103 (76.30)	800 (85.29)		
2-3 points	170 (15.84)	32 (23.70)	138 (14.71)		
**Type of incision**					
type II incision	1064 (99.16)	134 (99.26)	930 (99.15)	0.138	0.711
type III incision	9 (0.84)	1 (0.74)	8 (0.85)		
**Intraoperative blood loss (mL)**	261.01 ± 450.64	315.26 ± 410.60	253.20 ± 455.78	2.241	0.071
**Surgeon**				49.051	<0.001
Doctor A	70 (6.52)	6 (4.44)	64 (6.82)		
Doctor B	171 (15.94)	17 (12.59)	154 (16.42)		
Doctor C	452 (42.12)	47 (34.81)	405 (43.18)		
Doctor D	87 (8.11)	17 (12.59)	70 (7.46)		
Doctor E	51 (4.75)	4 (2.96)	47 (5.01)		
Doctor F	146 (13.61)	12 (8.89)	134 (14.29)		
Others	96 (8.95)	32 (23.70)	64 (6.82)		
**Surgical departments**				52.475	<0.001
Pancreatic center	1000 (93.20)	106 (78.52)	894 (95.31)		
Non-pancreatic centers	73 (6.80)	29 (21.48)	44 (4.69)		
**Intraoperative redosing**				2.723	0.256
Required but no redosing	733 (68.31)	94 (69.63)	639 (68.12)		
Required and redosing	40 (3.73)	8 (5.93)	32 (3.41)		
Not required	300 (27.96)	33 (24.44)	267 (28.46)		
**ERCP**				0.012	0.911
No	853 (93.94)	89 (93.68)	764 (93.97)		
Yes	55 (6.06)	6 (6.32)	49 (6.03)		
**Placement of biliary or pancreatic ductstents**				2.197	0.138
No	638 (70.26)	73 (76.84)	565 (69.50)		
Yes	270 (29.74)	22 (23.16)	248 (30.50)		
**SSI**				6.760	0.009
No	953 (88.82)	111 (82.22)	842 (89.77)		
Yes	120 (11.18)	24 (17.78)	96 (10.23)		

### Univariate and multivariate logistic regression analyses of AP > 24 h after pancreatic surgery

The univariate logistic regression analysis showed that age (OR = 1.02, 95% CI: 1.01–1.04), ASA score of 3–4 (OR = 1.78, 95% CI: 1.19–2.65), NNIS score of 2–3 (OR = 1.80, 95% CI: 1.16–2.78), other surgeon (OR = 5.33, 95% CI: 2.09–13.63), and surgery operated in non-pancreatic centers (OR = 5.56, 95% CI: 3.34–9.26) were risk factors for the AP > 24 h after pancreatic surgery, and the differences were statistically significant (all *p* < 0.05). Variables that were statistically different in the univariate analysis were included in the multivariate logistic regression analysis, which showed that the surgery operated in non-pancreatic centers (OR = 2.61, 95% CI: 1.04–6.54, *p* = 0.041) was independently associated with AP > 24 h after pancreatic surgery (Table 3).

**Table 3:** Univariate and multivariate logistic regression analyses of AP > 24 h after pancreatic surgery (*n* = 1,073).

Variable	Univariate analyses		Multivariate analyses	
	OR (95%CI)	*p* value	OR (95%CI)	*p* value
**Age (years)**	1.02 (1.01, 1.04)	0.002	1.01 (1.00, 1.03)	0.095
**Sex**				
Male	1			
Female	0.86 (0.60, 1.24)	0.416		
**Diabetes**				
No	1			
Yes	1.11 (0.70, 1.78)	0.652		
**Surgical category**				
Pancreaticoduodenectomy	1			
Distal pancreatectomy	0.83 (0.55, 1.25)	0.372		
Others	0.80 (0.48, 1.35)	0.410		
**Surgical approach**				
Non-endoscopic surgery	1			
Endoscopic surgery	0.81 (0.46, 1.42)	0.456		
**Surgical time (h)**	1.08 (0.97, 1.21)	0.173		
**Emergency**				
No	1			
Yes	0.95 (0.42, 2.14)	0.904		
**ASA score**				
1–2 points	1		1	
3–4 points	1.78 (1.19, 2.65)	0.005	1.41 (0.67, 2.93)	0.364
**NNIS score**				
0–1 point	1		1	
2–3 points	1.80 (1.16, 2.78)	0.009	1.15 (0.52, 2.53)	0.734
**Type of incision**				
Type II incision	1			
Type III incision	0.87 (0.11, 6.99)	0.894		
**Intraoperative blood loss (mL)**	1.00 (1.00, 1.00)	0.170		
**Surgeon**				
Doctor A	1		1	
Doctor B	1.18 (0.44, 3.12)	0.743	1.26 (0.47, 3.36)	0.647
Doctor C	1.24 (0.51, 3.01)	0.638	1.28 (0.52, 3.12)	0.595
Doctor D	2.59 (0.96, 6.98)	0.060	2.36 (0.87, 6.42)	0.093
Doctor E	0.91 (0.24, 3.40)	0.886	0.92 (0.24, 3.45)	0.890
Doctor F	0.96 (0.34, 2.66)	0.9306	1.01 (0.36, 2.81)	0.992
Others	5.33 (2.09, 13.63)	0.001	2.64 (0.80, 8.70)	0.110
**Surgical departments**				
Non-pancreatic centers	5.56 (3.34, 9.26)	<0.001	2.61 (1.04, 6.54)	0.041
Pancreatic center	1		1	
**Intraoperative redosing**				
Required but no redosing	1			
Required and redosing	1.70 (0.76, 3.80)	0.196		
Not required	0.84 (0.55, 1.28)	0.418		
**ERCP**				
No	1			
Yes	1.05 (0.44, 2.52)	0.911		
**Placement of biliary or pancreatic duct stents**				
No	1			
Yes	0.69 (0.42, 1.13)	0.140		

### Comparison of the rates of AP in different surgical departments >24 h after pancreatic surgery

**Figure 4:**
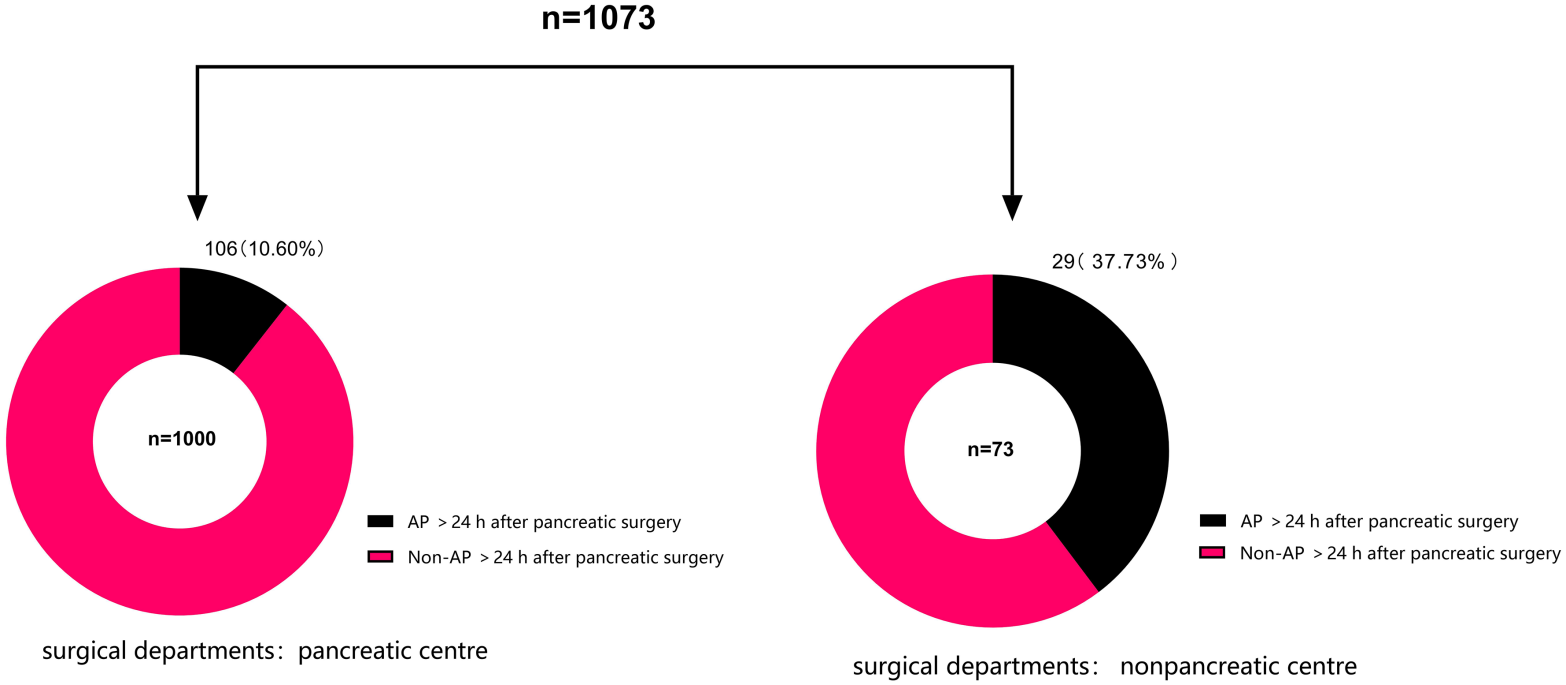
Comparison of postoperative prophylactic antimicrobial use rates after pancreatic surgery in different surgical units.

Among 1,073 patients, the rate of AP > 24 h after pancreatic surgery operated in non-pancreatic centers (37.73%, 29/73) was much greater than that in pancreatic centers (10.60%, 106/1,000), with the odds of using antimicrobial drugs 24 h after surgery being 2.61 times that in pancreatic centers (Figure 4).

### Association between extended postoperative antibiotic prophylaxis beyond 24 h and SSI after pancreatic surgery

We evaluated the association between extending postoperative antibiotic prophylaxis beyond 24 h and SSI after pancreatic surgery (Table 4). In the fully adjusted model, extended AP was not associated with a lower risk of SSI (OR 1.67, 95% CI 0.997–2.79; *p* = 0.053), indicating that postoperative antibiotics beyond 24 h were not associated with a decreased occurrence of SSI.

**Table 4:** Relationship between postoperative AP beyond 24 h and SSI following pancreatic surgery.

Exposure	Unadjusted estimate^*^		Model 1^*^	Adjusted estimate^1^	Model 2^*^	Adjusted estimate^2^
	OR (95% CI)	*p*	OR (95% CI)	*p*	OR (95% CI)	*p*
**SSI**						
AP > 24 h after pancreatic surgery						
No	1		1		1	
Yes	1.90 (1.16, 3.09)	0.010	1.84 (1.12, 3.03)	0.015	1.67 (0.997, 2.79)	0.053

AP, antibiotic prophylaxis; SSI, surgical site infection; OR, odds ration; CI, confidence interval; ASA, American Society of Anesthesiology; NNIS, National Nosocomial Infections Surveillance.

1 Model 1 was adjusted for age, sex, and diabetes.

2 Model 2 was adjusted for age, sex, diabetes, surgical category, surgical approach, surgical time, emergency, ASA score (0 and 1), NNIS score 1 (0 and 1 point) and (2 and 3 points).

* The sample size used for unadjusted estimate, Model 1, and Model 2 is all 1,073.

## Discussion

Although current guidelines recommend discontinuing AP within 24 h for most procedures, a significant “know-do” gap persists in clinical practice (18). A previous study (19) showed that only approximately 22% of patients’ AP procedures completely complied with all the guideline recommendations, and the most common types of violations were the duration of prophylaxis (14%) and appropriate drug selection (35%). This retrospective study of 1,073 patients yields two primary insights regarding AP in pancreatic surgery. First, we observed a substantial gap between guidelines and practice, with 12.58% of patients receiving AP > 24 h. Second, and most importantly, we identified a distinct organizational disparity: pancreatic surgery performed in non-pancreatic centers was an independent predictor associated with prolonged AP usage, whereas extending AP beyond 24 h was not associated with a decreased risk of SSI. These findings underscore the need to enhance implementation of current guidelines in non-specialized surgical units, where barriers to AP compliance require targeted educational and monitoring interventions to ensure adherence to evidence-based recommendations.

Our findings revealed that despite guideline recommendations, the average cumulative duration of AP was 6.24 ± 4.09 days, with the 4–7 day range accounting for 41.48% of the extended usage group. These rates are notably higher than those reported by Vangelis et al. (20) (7.2% for > 24 h and 7.6% for 4–7 days). This discrepancy may stem from the fact that the study by Vangelis et al. encompassed a broader range of procedures, including general, orthopedic, gynaecologic, and cardiac surgeries. The high incidence of postoperative complications of pancreatic surgery is the reason why many hospitals have a high proportion of patients treated with AP after pancreatic surgery, especially pancreaticoduodenectomy (21). However, there is still some controversy about whether the AP treatment time should be limited to 24 h after pancreatic surgery. Studies (22) have reported that a preoperative dose of antibiotics may be sufficient for preventing SSI in patients undergoing pancreatic surgery. Some scholars have suggested that AP use should be limited to 24 h after surgery, while others have suggested that AP could be used for a longer time, especially in high-risk patients or patients who have preoperative biliary drainage (21, 23, 24). One of the main shortcomings of long-term use of AP is that selective antibiotic pressure at the individual and population levels may lead to antibiotic resistance. The effects of long-term prophylaxis on antibacterial resistance and the gut microbiota should be evaluated (12). Moreover, our previous study showed that prophylaxis use for 48 h after pancreatic surgery cannot reduce the SSI incidence (4). The effect of long-term AP for the prevention of SSIs after pancreatic surgery needs to be further evaluated in sufficient, effective, randomized controlled trials. Although a small proportion of extensions may be clinically justified in complex pancreatic procedures (e.g., preoperative biliary drainage), this finding suggests that there is still room to optimize adherence to short-course prophylaxis. In routine practice, achieving 100% adherence is unlikely to be realistic; instead, quality-improvement efforts should aim to keep the vast majority of patients within 24 h of AP, while allowing carefully justified exceptions.

Chinese guidelines recommend the administration of first- and second-generation cephalosporin or ceftriaxone with or without metronidazole, as well as cephalomycin, as perioperative AP in hepatopancreatobiliary surgery (11). Regarding antimicrobial selection in this study, third-generation cephalosporins and enzyme inhibitor combinations were the predominant agents used across all time periods. The regimens used were similar to the third-generation cephalosporins combined with anti-anaerobic drugs used in the study of Wang et al. (25), and somewhat different from the routine use of second-generation cephalosporins for preoperative AP (26). This likely reflects the specific microbial ecology of pancreatic surgery, which involves potential contamination from intestinal flora dominated by Gram-negative bacilli. Additionally, postoperative decisions to continue or switch antibiotics may be driven by intraoperative bile culture results, leading to individualized regimens. However, clinicians must weigh this against the risk of broad-spectrum β-lactam antibiotics severely disrupting the gut microbiota (27).

Understanding the determinants of prolonged AP is crucial for designing effective stewardship interventions. A key finding of this study is that pancreatic surgery performed in a non-pancreatic centers was an independent predictor associated with AP > 24 h (OR = 2.61, 95% CI: 1.04–6.54). The probability of extended AP usage in non-pancreatic centers was more than double that in specialized pancreatic centers (37.73% vs. 10.60%). This disparity highlights a significant target for quality improvement. Non-specialist surgeons, who perform pancreatic procedures less frequently, may rely on extended AP as a perceived “safety net” to mitigate risks associated with longer operative times or greater blood loss (18). These observations further alert us to potential deficiencies beyond AP duration in non-pancreatic centers, warranting comprehensive investigation of preoperative AP timing and appropriateness of antimicrobial agent selection in perioperative prophylaxis. These findings also have significant practical implications: simply publishing guidelines is insufficient. Hospitals should implement targeted interventions for non-specialized departments, such as mandatory consultation with infectious disease specialists for pancreatic cases, specific electronic order sets that auto-stop antibiotics at 24 h unless an infection is documented, and enhanced multidisciplinary training. Standardizing management protocols across all departments performing pancreatic surgery is essential to bridge the gap in care quality. Multidisciplinary collaborative management (13) has been shown to be effective in improving the compliance rate of AP guidelines (36.6% vs. 57.9%), including complying with the recommended duration of postoperative prophylaxis (71% vs. 80.1%), which provides direction and confidence for our subsequent management work.

Although Model 2 adjusted for traditional confounding factors, other important confounders may not have been adequately controlled. Specifically, doctors’ decisions to prolong AP may rely on preoperative/intraoperative pathogen information or perceived infection risk signals such as pathogenic organisms identified from preoperative bile drainage, or intraoperative findings suggesting infection risk. However, it is important to emphasize that AP differs fundamentally from empiric therapeutic antibiotics. Extended prophylaxis should not be conflated with therapeutic antibiotic courses warranted by documented or highly suspected infection. Considering the evidence from this study and previous studies (4), it is advisable in clinical practice to discontinue AP within 24 h in routine cases, and reserve therapeutic antibiotics for confirmed or highly suspected infection.

This study has several limitations. First, as a single-center study, the findings may not fully generalize to other institutions with different resistance patterns. Second, the mortality rate in our cohort was very low and detailed mortality data were not systematically available for all patients, so we were unable to evaluate the association between extended postoperative AP and mortality. Future multicenter prospective studies are needed to further validate these findings.

## Conclusion

In summary, a substantial proportion of patients (12.58%) continued to receive AP > 24 h after pancreatic surgery, deviating from current guidelines. Surgery performed in non-pancreatic centers was identified as an independent predictor associated with prolonged AP usage, highlighting a disparity in antibiotic stewardship between specialized and non-specialized departments. Crucially, extending AP beyond 24 h was not associated with a decreased risk of SSI after adjusting for confounders. These findings suggest that for the vast majority of patients, prolonged prophylaxis provides no additional benefit and may contribute to unnecessary antibiotic exposure. Therefore, targeted quality improvement initiatives are urgently needed to standardize antibiotic management protocols, specifically aiming to align practices in non-specialized units with those in high-volume pancreatic centers.

## Data Availability

The original contributions presented in the study are included in the article/supplementary material, further inquiries can be directed to the corresponding authors.

## Footnotes

1
www.empowerstates.com
